# The Response of Phagocytes to Indoor Air Toxicity

**DOI:** 10.3389/fimmu.2017.00887

**Published:** 2017-07-28

**Authors:** Liisa K. Vilén, Janne Atosuo, Esa-Matti Lilius

**Affiliations:** ^1^Department of Biochemistry, University of Turku, Turku, Finland

**Keywords:** neutrophils response, mold, indoor air pollution, toxicity tests, immuno response

## Abstract

This perspective presents a viewpoint on potential methods assessing toxicity of indoor air. Until recently, the major techniques to document moldy environment have been microbial isolation using conventional culture techniques for fungi and bacteria as well as in some instances polymerase chain reaction to detect microbial genetic components. However, it has become increasingly evident that bacterial and fungal toxins, their metabolic products, and volatile organic substances emitted from corrupted constructions are the major health risks. Here, we illustrate how phagocytes, especially neutrophils can be used as a toxicological probe. Neutrophils can be used either *in vitro* as probe cells, directly exposed to the toxic agent studied, or they can act as *in vivo* indicators of the whole biological system exposed to the agent. There are two convenient methods assessing the responses, one is to measure chemiluminescence emission from activated phagocytes and the other is to measure quantitatively by flow cytometry the expression of complement and immunoglobulin receptors on the phagocyte surface.

## Introduction

Indoor air problem is a tremendous health hazard, especially when a living building or working place is infested with toxic molds (1). Although microbial communities on surfaces do not directly correlate with the health trouble of the occupants, in many countries conventional techniques to document moldy environment due to dampness has been traditionally based on culture techniques using conventional isolation media and quantitation of colony forming units per, e.g., cubic meter (2). However, well-designed and well-conducted international so-called health effects of indoor pollutants: integrating microbial, toxicological, and epidemiological approaches studies proved that quantities of molds *per se* were not as good health correlates as the level of microbial markers (3–5). Indeed, the toxicity of molds and other toxic compounds emitted from damaged surfaces had a detrimental health effect as unambiguously shown in teaches in mold-infected schools (6). Therefore, it is imperative to adopt new thinking to search and exploit novel methods that will be robust enough, inexpensive, and reliable to be implemented into routine. Indoor air toxicity detection instead of colony detection is the future of environmental research.

Mycotoxins are usually analyzed with mass-spectrometry-based techniques that are coupled with pre-separation by a gas or liquid chromatography. These methods allow for the highly sensitive and accurate determination of tested samples. They are, however, expensive and time-consuming, taking from days up to weeks to obtain the results, and still providing only the detection of a single compound or a group of structurally related compounds at any given time (7). Moreover, the conventional methods do not take into account the possible synergistic effects of the mycotoxins and other microbial structure components as well as toxic compounds emitted from damaged surfaces which may enhance the toxicity. New technologies are becoming available that may enable the better assessing of the total toxicity. We have developed a test system which assesses rapidly and cost-efficiently the total indoor air toxicity using the *Escherichia coli*-lux method (8). While correlating well with the building-related symptoms of users, this assay can be criticized for using prokaryotic cells as probes. Below, we present ideas and directions of research based on mammalian neutrophils that could be exploited as probe cells for toxicity studies.

## Principles of Phagocyte Activation

The process of phagocytosis is recently reviewed by Gordon (9). The generation and measurement of phagocyte chemiluminiscence (CL) are thoroughly described by Lilius and Marnila (10). Briefly, the activation of phagocytes produce electronically excited states, which on relaxation to the ground state emit photons, referred to as phagocyte CL. The commonly used activators include various opsonized or unopsonized particles and immune complexes. “In the opsonization process particles attach to complement compounds and immunoglobulins. Opsonized antigens are recognized partly by the complement receptor CR3, partly by the complement receptor CR1, and partly by FcγRII and FcγRIII receptors, which bind to the Fc portion of the IgG molecules attached to antigens.

Phagocytic cells are generally isolated from blood treated with anticoagulants. Leukocytes obtained after erythrocyte sedimentation are normally sufficient without further separation. Phagocytic cell activities can also be measured in the *ex vivo* state simply by diluting the whole blood enough to get rid of the inhibitory amounts of plasma and red cells. The *ex vivo* cells are, however, not necessarily in the same functional state as the cells after isolation steps where activation processes may take place. Luminol amplifies the CL emission by the factor of 10^3^–10^4^ and it has been shown to be oxidized in the myeloperoxidase reaction. When using luminol in the millimolar range, one needs less than a thousand phagocytic cells (as in the case in whole blood tests) to get reliable signals. The number of isolated cells used in routine tests varies, generally around 10^5^. Hank’s balanced salt solution (HBSS) is the most frequently used buffer. Luminometers with strict temperature controls, multiple sample capabilities (up to 96 in microtiter plate readers) and computerized data processing are the instruments of choice” (10).

## Measurement of *In Vitro* Toxicity

Routinely, *in vitro* toxicity testing was made as luminol-amplified CL assay by adding 25 µl of opsonized zymosan suspension (20 mg/ml) in HBSS buffer supplemented with gelatin (1 mg/ml) (gHBSS), 20 µl of luminol (10 mM in borate buffer, pH 9.0), and 100 µl of leukocyte suspension (varying number of neutrophils depending on the isolation method) to the wells of a white 96-well microtiter plate. Finally, toxic samples in various concentrations were added. The final reaction volume was 200 µl.

**Figure 1:**
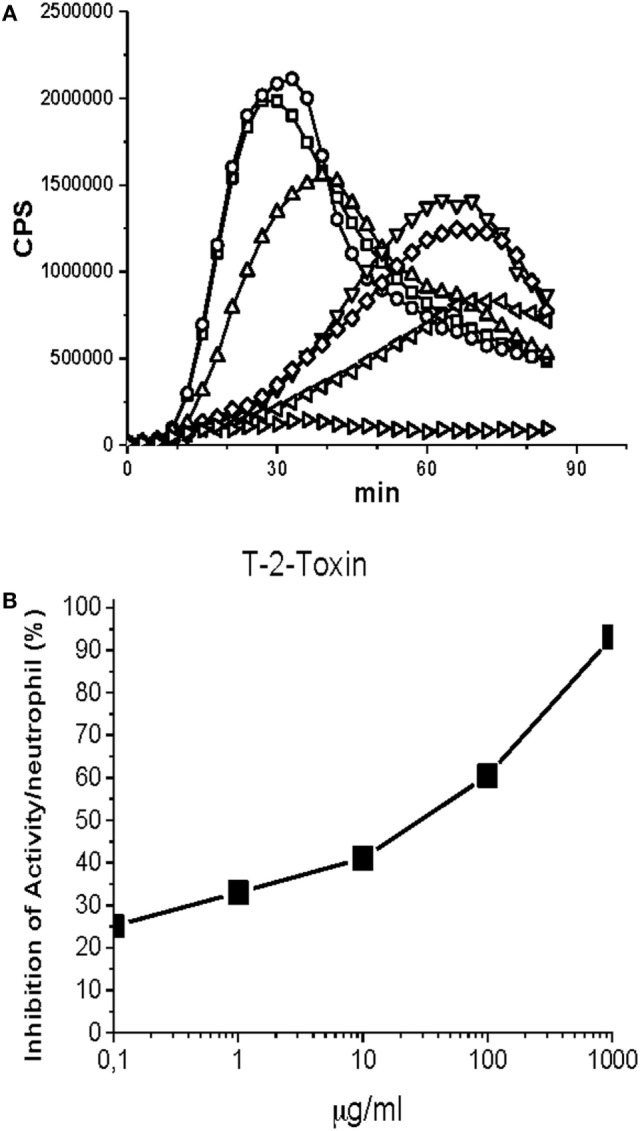
Effect of T-2 toxin on neutrophil chemiluminescence was induced by opsonized zymosan. The reaction mixture contained 25 µl of opsonized zymosan suspension (20 mg/ml) in Hank’s balanced salt solution buffer supplemented with gelatin (1 mg/ml), 20 µl of luminol (10 mM in borate buffer, pH 9.0), and 100 µl of neutrophil suspension (50,000 cells). Finally, toxic samples in various concentrations were added. The final reaction volume was 200 µl. T-2 toxin: 0 µg/ml (□), 0.01 µg/ml (○), 0.1 µg/ml (▵), 1 µg/ml (▿), 10 µg/ml (◊), 100 µg/ml (◃), 1,000 µg/ml (▹). **(A)** The kinetic curves of the chemiluminiscence (CL) emission of neutrophils. **(B)** Inhibition of the peak CL emission of neutrophils by the T-2 toxin. **(A)** A strong luminol-amplified CL signal peaking at about 30 min was detected when opsonized zymosan was added to the reaction mixture containing 5 × 10^4^ neutrophils. When, in addition to opsonized zymosan, also toxic samples were added the CL signal was dose-dependently reduced. EC50 value was determined from the dose curve where the CL signal was reduced 50%. T-2 toxin from *Fusarium* species appeared to have an EC50 value of 30 µg/ml calculated from peak CL values **(B)**. EC50 is the concentration of a toxin that kills 50% of the bacterial cells. CPS is counts per second registered by the luminometer.

The CL signals of the microtiter plate wells were continuously recorded 0.5 s/well for 200 min in Hidex Sense multimode reader (Hidex Ltd., Turku, Finland) at 37 C. Three parallel wells were prepared from every reaction mixture. The background signal was measured from a well containing only the buffer and this reading was subtracted from the readings of the experimental wells. Figure 1 illustrates the principles of this technique.

The other tested mycotoxins deoxyvalenol, moniliformin, antimycin A, and chloramphenicol had EC50 values ranging from 20 µg/ml to a few hundreds of micrograms per milliliter. The same mycotoxins gave similar or roughly 10-fold lower EC50 values in our *E. coli-*lux toxicity test (8). It should be noted that cytochalasin D was very weakly toxic in *E. coli*-lux toxicity test while chloramphenicol, being very toxic in *E. coli*-lux toxicity test, was weakly toxic in the neutrophil toxicity test. A dust sample (11) from a moisture-damaged object had EC50 values of 72 and 15 µg/ml in neutrophil toxicity test and *E. coli*-lux toxicity test, respectively.

By careful choosing the reaction conditions, one is able to differentiate between the binding and ingestion phases of phagocytosis in CL measurements. In these experiments, the ingestion of zymosan by neutrophils was measured flow-cytometrically as described by Nuutila and Lilius (12). Cytochalasin D inhibited the ingestion of opsonized (0.5% serum) and non-opsonized zymosan dose-dependently. The CL response induced by 25 µg zymosan consisted of two peaks with peak times of about 10 and 90 min, and 10 and 65 min in the absence and the presence of serum (0.5%), respectively. The second peak in the CL response is clearly the ingestion peak since cytochalasin D gradually inhibited it. The first peak is then the adhesion peak, which increases with increasing the concentration of cytochalasin D. The ingestion is dependent on the cytoskeleton and thus on ATP produced by mitochondria. We anticipate that well-known fungal and bacterial toxins affecting mitochondria such as antimycin A (13) reduce the ingestion phase but not the binding phase of phagocytes being expressed as a decrease of the second peak of the CL response.

## Quantitation of Fungal Spore Antibodies

Fungal and bacterial spores are one of the main predisposal elements in contaminated indoor air (14–17). The virulence of many pathogens relates in part to their ability to evade phagocytosis by the virtue of certain surface antigens. The function of serum antibodies is to react with these antigens and make the microorganisms more susceptible to ingestion by neutrophils. When serum is incubated with spores, and if specific antibodies are present, the spores are rapidly and efficiently opsonized and phagocytosed by neutrophils. Thus, when this reaction is measured as a CL emission the exposure of an individual to spores is manifested as an elevated CL peak compared with the non-exposed controls. This CL emission correlates with the level of antibodies. The system is functional with both isolated cells and with the whole blood dilutions in both heterologous and autologous systems (18).

The spore assay was operated as described in the section “The measurement of in vitro toxicity” but instead of opsonized zymosan, test serum (1%) and intact spores (~10^10^) were added. *Streptomyces albus* spores were cultured and harvested from the mannitol salt agar and *Aspergillus versicolor* spores from malt agar by scraping and by diluting them into the distilled water. This suspension was then sonicated. Spores were isolated by filtration trough the cotton wool and re-suspended in 20% glycerol (19). When *S. albus* or/and *A. versicolor* spores and test serums were introduced to neutrophils the CL emission was significantly higher in individuals exposed to these microbes in damaged buildings (data not shown).

The autologous system utilizing neutrophils from the fingertip whole blood enables the development of *in situ* quick test systems to be operated with a portable luminometer.

By comparison, spore specific IgG and IgM antibody titers were assayed using enzyme-linked immunosorbent assay with intact spores as antigens (16, 20). Spores were fixed to the solid face of the microtiter wells, incubated with the test serum, and then with the anti-human immunoglobulin tagged with enzyme horseradish peroxidase. The enzyme activity from the solid phase was related to the bound antibody.

Serum samples of the exposed individuals from the microbe damaged buildings expressed elevated serum spore specific IgG and IgM antibody titers but IgM was in better correlation with the increased CL response from the same test serums.

It is noteworthy that according to literature (20) and our study the fungal and bacterial spore antigens may share common structures since there is substantial cross-reactivity in antibody responses regardless of the origin of the spore. If this is true, a single test antigen instead of panels containing multiple microbe antigens can be used.

## Estimation of Toxicity *In Vivo*

We are aiming to collect human blood samples of the users of moisture-damaged buildings for establishing a biomonitoring assay for the toxic effects on exposed users. This assay is based on the measurement of the capability of phagocytes to emit photons when stimulated with opsonized zymosan. This has been shown to reflect remarkably well the pathophysiological state of the host. In many cases even the magnitude of the stress, the presence of pathogen in the body, or the activity of the disease can be estimated (10). Therefore, we believe that neutrophils can be utilized as a toxicological probe acting as an indicator of the whole biological system exposed to the agent. Here we show that the concept is indeed valid with test animals. How it will work with humans remains to be elucidated later.

Previous studies have shown that moniliformin is acutely toxic to rats with an LD50 cut-off value of 25 mg/kg bw (21). Here are the results of a subacute oral toxicity study. Rats were daily exposed to moniliformin of low doses from 0 to 15 mg/kg bw for 28 days. Two satellite groups were kept alive for an additional 14 days without treatment to detect possible delayed effects and to follow up recovery. The neutrophil CL measurements revealed the toxic effect of moniliformin on the rat innate immunity.

The phagocytic activity of the rat neutrophils was dramatically reduced in all dose groups and did not recover. “Even the lowest dose (3 mg/kg bw) caused a substantial decrease in the neutrophil activity reducing the final activity by 70% of the initial activity. Moreover, the decrease in neutrophil activity continued in the satellite groups subsequent to the cessation of moniliformin exposure, with a mean activity of 28 ± 18% in the final samples, compared with the negative control group. Also the neutrophil activity of the final samples of the negative control group was reduced by 29% from the initial samples. The reduction in the control group was most likely derived from stress caused by housing in metabolic cages, handling, blood collection, and daily i.g. administration of the vehicle by gavage. In spite of the reduced phagocytic activity of neutrophils, the number of neutrophils and total leukocyte counts in the blood samples of the dosed rats remained normal, suggesting that moniliformin has a functional effect on myeloid cells, rather than a lymphoid one. Additionally, the lymphoid organ weights (thymus, spleen) were unaffected, excluding dystrophic, and dysplastic effects.” (21).

## Algorithms of Expression of Different Surface Receptors on Phagocytes

Do the infectious and other inflammatory diseases or exposure to indoor air pollutants induce alterations in the expression of the opsonin receptors of phagocytes?

Receptor expression measurements are described in Ref. (22). Briefly, erythrocytes were lysed and leukocytes were separated by centrifugation. Leukocytes (3 × 10^5^) were incubated in 50 µl of gHBSS with receptor-specific monoclonal antibodies (0.4 µg) for 30 min at +4 C. A relative measure of receptor expression was obtained by determining the mean fluorescence intensity of 5,000 leukocytes. In the case of neutrophil FcγRI, the percentage of fluorescence positive cells (%) was also determined. Measurement of leukocyte receptor expression was performed using fluorescently (FITC or PE) labeled receptor-specific monoclonal antibodies.

We have performed a few studies where we have measured the receptor expression in various patient groups (23–26). The summary of the results of these studies is presented in Table 1. In monocytes, all the receptors were upregulated in bacterial and viral infections. In neutrophils, CR1, CR3, FcγRI, and FcγRII were upregulated, while FcγRIII was downregulated in bacterial infections. CR1 and FcγRII were downregulated, while CR3 and FcγRI were upregulated in viral infections. These results led us to conclude that the receptor expression could be used as a basis for the differential diagnosis of bacterial and viral infections (22). Whether the exposure to molds causes a specific pattern in opsonin receptor expression will be studied in a near future if resources are available.

**Table 1:** Receptor expression changes in various diseases compared with health controls.

Receptor	Bacterial infection	Viral infection	Kidney cancer	Atopic dermatitis
**Neutrophils**				
CR1/CD35	+++	(−)	No change	+
CR3/CD11b	+++	+	++	(+)
FcyRI/CD64	+++	+++	(+)	No change
FcyRII/CD32	+	(−)	No change	(+)
FcyRIII/CD16	(−)	No change	No change	(−)
**Monocytes**				
CR1/CD35	+++	++	+	(+)
CR3/CD11b	+++	++	+++	(+)
FcyRI/CD64	+++	+++	+	(−)
FcyRII/CD32	(+)	No change	++	(+)

*The +/− without parentheses indicates a significant increase/decrease in the expression of receptor in question compared with healthy control. The +/− in parentheses indicates an insignificant increase/decrease in the expression of receptor in question compared with healthy control. + or − = 0–50% increase or decrease compared with healthy control, ++ = 50–100% increase compared with healthy control, and +++ = more than 100% increase compared with healthy control*.

## Conclusion

This perspective highlights potential of neutrophils as a toxicological tool for studies of indoor air toxicity. Neutrophils are mammalian cells, therefore any toxicity imposed on them can be easily to extrapolated on the whole human body. They can be used either as probe cells, directly exposed to samples collected from damaged buildings or they can act as indicators of toxic effects on humans exposed to the toxic compounds of damaged buildings. The future shows whether the receptor expression measurements from leukocytes bring additional evidence for the indoor air toxicity assessments.

Part of this study is presented in the Indoor Air 2016 Conference in Ghent, Belgium, July 3–8, 2016 (27).

## Author Contributions

E-ML: project manager, planning the research/laboratory tests, analyzing the data. JA: execution and planning of the laboratory experiments, analyzing the data. LV: execution and planning of the laboratory experiments, analyzing the data.

## Conflict of Interest Statement

The authors declare that the research was conducted in the absence of any commercial or financial relationships that could be construed as a potential conflict of interest.
